# Pharmacological Studies in Hospitalized COVID-19 Patients in Belgium: We Could Do Better

**DOI:** 10.3390/v14071427

**Published:** 2022-06-29

**Authors:** Maya Hites, Jean-Louis Vincent

**Affiliations:** 1 Clinic of Infectious Diseases, HUB-Erasme Hospital, Université Libre de Bruxelles, 1070 Brussels, Belgium; maya.hites@erasme.ulb.ac.be; 2 Department of Intensive Care, Erasme Hospital, Université Libre de Bruxelles, 1070 Brussels, Belgium

**Keywords:** Belgium, adaptive platform trials, severe acute respiratory syndrome coronavirus 2 (SARS-CoV-2)

## Abstract

Belgium has actively participated in clinical research on severe acute respiratory syndrome coronavirus 2 (SARS-CoV-2) since the beginning of the pandemic to help identify effective and safe treatments for COVID-19. The objective of this review is to provide a picture of the clinical studies carried out in hospitalized patients with COVID-19 in Belgium. We collected data on all randomized, interventional trials in patients with COVID-19 that were registered on two recognized clinical trial registers, started enrollment before 31 December 2021, and included at least one patient in a Belgian center. Data were collected concerning the therapies investigated and the nature of the trials performed. Thirty-three hospitals (32% of all Belgian hospitals) participated in at least one of 28 trials (13 sponsored by the industry and 15 by academic centers) on therapeutics for COVID-19 in hospitalized patients: 7 (25%) evaluated antivirals, 17 (61%) immunomodulators, 2 (7%) anti-coagulants, and 1 (3%) nitric oxide to improve respiratory function. Nineteen (68%) were phase II trials. Only three (11%) of the trials were international platform trials. Despite numerous trials, less than 3% of all Belgian patients hospitalized with COVID-19 participated in a clinical trial on therapeutics. As in many other countries, more efforts could have been made to avoid running small, under-powered, mono- or bicenter trials, to create better collaboration between the different Belgian hospitals, and to participate in more international clinical trials, and more specifically in adaptive, platform trials.

## 1. Introduction

The world was taken by surprise by the severe acute respiratory syndrome coronavirus 2 (SARS-CoV2) pandemic, which began in December 2019. It has caused significant economic and social disruption, as well as high morbidity and mortality rates worldwide. Indeed, as of the 8 June 2022, over 530 million SARS-CoV-2 infections have been diagnosed worldwide, causing saturation of healthcare services, numerous lockdowns, and over 6.2 million deaths attributed to coronavirus disease 2019 (COVID-19) since the beginning of the pandemic [1]. When this novel virus first emerged, clinicians were obliged to provide care to patients affected by a disease that they knew little about. In this light, clinical trials were, and remain, essential to provide guidance to clinicians on how to best treat patients with COVID-19.

In 2014, before the SARS-CoV-2 pandemic, West Africa had to deal with an Ebola epidemic; over 30,000 individuals developed the disease, and mortality was high. Many therapies were tested, but few comparisons were made to a placebo or control group. Indeed, randomized controlled trials were launched too late in the epidemic, and by the time the trials had authorizations to begin, there were few cases to include [2]. At the end of the epidemic, no conclusions could be drawn concerning the efficacy, nor the security, of most molecules tested. After many discussions within the World Health Organization (WHO), some decisions were reached: there is an ethical obligation to carry out clinical trials when an epidemic with significant health consequences occurs and for which no effective treatment is known, and research needs to be an integral part of the public health response to the epidemic [3]. Furthermore, platform trial designs were identified to be well suited to ethically and efficiently identify effective therapies in the setting of an emergency outbreak [4].

Coming back to the SARS-CoV-2 pandemic, much research has been carried out but, on a global level, it has not been sufficiently coordinated and, once again, much energy has been wasted on clinical trials without placebos or controls, or on under-powered trials with small sample sizes, particularly in the early days of the pandemic [5]. There have been coordinated efforts to launch large-scale platform trials, but the uptake has not been universal.

Belgium is a rich European country with a population of around 11 million inhabitants. Health expenditure in 2017 was 10.3% of the gross domestic product (GDP), corresponding to the eighth highest in the WHO European region at the time [6]. As in many countries worldwide, Belgium was significantly affected by the SARS-CoV-2 pandemic, and has actively participated in research on SARS-CoV-2 since the beginning of the pandemic. It is now time to evaluate the research carried out with a critical eye, to identify strong points in the research efforts, but also to identify where improvements can be made. Research can be very varied, from fundamental science to clinical trials. This evaluation will only focus on therapeutic clinical trials for COVID-19 performed in Belgium in hospitalized patients.

## 2. Materials and Methods

### 2.1. General Information Concerning the Belgian Healthcare System

Belgium is a federal state with parliamentary democracy. Jurisdiction over health policy and regulation of the healthcare system are divided among the federal state and the federated entities, made up of three regions based on geography (Flanders in the north, Wallonia in the south, and the Brussels region in the center) and three communities based on language (Flemish, French, and German). Although there has been a transfer of additional healthcare competencies from the federal state to the federal entities since 2014, the federal state remains competent for matters that concern all Belgians. Therefore, the response to the SARS-CoV-2 pandemic was partially a federal responsibility.

There are 103 hospitals with a total of 34,962 accredited acute-care beds in Belgium: 79 (76.7%) are general hospitals, 17 (16.5%) are general hospitals with a university link, and 7 (6.8%) are university hospitals. The number of beds/100,000 inhabitants is greatest in the Brussels capital region, with 673.8 beds/100,000 inhabitants in 2019, followed by 458.7 beds/100,000 inhabitants in the Flanders region and 379.8 beds/100,000 inhabitants in the Wallonia region [6]. 

### 2.2. Data Sources

We collected data on all randomized, interventional trials for patients diagnosed with COVID-19 in Belgium that were registered on www.clinicaltrials.gov and/or www.clinicaltrialsregister.eu, started enrollment before 31 December 2021, and included at least one patient in a Belgian center. The search terms used were “Belgium” and “COVID-19”. We limited the end search date to the 31 December 2021, as we consider this period to be the “pre-Omicron era” in Belgium. The epidemiology of hospitalizations changed markedly after the Omicron variant became the dominant circulating virus, with many polymerase chain reaction (PCR) SARS-CoV-2-positive patients hospitalized for indications unrelated to their infection. 

Data were collected concerning the therapies investigated and the nature of the trials performed: whether they were observational or randomized, whether there was a control group or placebo used, whether sponsorship was academic or from the industry, and whether they were multicenter and/or international. The hospitals that participated in each trial and their characteristics, in terms of their geographical location (Brussels, Flanders or Wallonia), their acute bed capacity, and their classification (general hospital, general hospital with a university link, or university hospital), were also recorded. 

Other data collected included the number of patients planned for inclusion (if still recruiting) or actually included (if completed or terminated) in Belgium for each of the trials, the number of participants included in the trials financed by the Belgian Healthcare Center for Knowledge (KCE; the federal agency responsible for financing clinical trials of public health importance from the beginning of the pandemic until the 31 December 2021), the time in days needed to obtain national regulatory and ethics approval for each trial, and the dates of start and completion (if relevant) for each trial. If the study or an arm of a study was completed or terminated, information concerning publication or communication of the data, as well as whether the data had been shared for use in at least one meta-analysis, was also recorded. We also evaluated whether the results of the trials had an impact on international therapeutic guidelines concerning treatment of hospitalized patients with COVID-19. Results were considered to have had an impact when the trial or a meta-analysis using data from the trial was cited as a reference in an international guideline on therapeutics for hospitalized patients with COVID-19.

Concomitant to the cross-sectional study, data were recorded concerning the number of hospitalizations for COVID-19 in Belgium from the beginning of the pandemic until the 31 December 2021, as well as the Belgian consumption of remdesivir from the beginning of the pandemic until the 31 January 2021 (the DisCoVeRy trial stopped the arm studying standard of care (SoC) versus remdesivir plus SoC on the 20 January 2021). Finally, information concerning the funding provided at the Belgian national level to support clinical trials on therapeutics for COVID-19 in the hospital setting was also obtained. 

Data are reported as numbers and percentages for non-continuous variables, and as median (minimum–maximum) for continuous variables without a normal distribution. 

Ethics approval was not required because all data are publicly available.

## 3. Results

Belgian hospitals participated in a total of 28 trials (13 sponsored by the industry and 15 by academic centers) on therapeutics for COVID-19 in hospitalized patients from the beginning of the pandemic until the 31 December 2021. The trial characteristics are provided in Table 1 and Table 2, for those sponsored by the industry and by academic centers, respectively. 

The therapeutics evaluated in the trials were antivirals (7 trials (25%)), immunomodulators (17 (61%)), anti-coagulants (2 (7%)), and nitric oxide to improve respiratory function (1 (3.4%)). Most of the trials (19 (68%)) were phase II trials; two (7%) were phase I, eight (29%) were phase III, and one (4%) was a phase IV trial (several trials were also reported as phase I/II or phase II/III). Thirteen (46%) trials were only carried out in Belgium, and five (18%) were mono- or bicenter trials. Only three (11%) of the trials were international platform trials. Twenty of the trials or arms of a trial have been completed or terminated. Only 10 (36%) of the trials have communicated their results. Four (14 %) of the trials have provided data for meta-analyses [7,8,9,10]. Results from four (14%) of the trials had an impact on international therapeutic guidelines concerning treatment of hospitalized patients with COVID-19. All four of these trials were multicenter (minimum of 16 sites), and two of the trials were international, adaptive, platform trials. 

A total of 33 hospitals (32% of all hospitals in Belgium) participated in at least one of these clinical trials. Belgian hospital participation was greatest in the Flanders region (19 (58%)); eight hospitals (24%) were from Wallonia, and seven (21%) from the Brussels region. All university hospitals, all hospitals with a university link, and nine (1.3%) general hospitals participated in at least one of the trials. Overall, university hospitals participated in a median of 6(4–8) trials, and the hospitals with a university link participated in a median of 1(1–7) trials.

The total number of Belgian patients planned for inclusion (for trials not yet completed) or actually included (for completed trials or trial arms) in all trials initiated before 31 December 2021 was 2798. A total of 1351 participants were included in the four KCE funded trials by 33 participating Belgian centers. 

During this same period, Belgium recorded 2,125,876 SARS-CoV-2 infections, 96,770 hospitalizations, and 28,403 deaths due to COVID-19 [11]. A total of 12,507 doses of remdesivir, corresponding to 2501 treatments of 5-day duration, were delivered to hospitals for use between 1 September 2020 and 31 January 2021 (data communicated from the AFMPS (Federal Agency for Medicines and Health Products)). 

A total of EUR 7.5 million was provided to finance the four KCE trials [12]. However, financing was ended prematurely for the DisCoVeRy trial when the trial began evaluating AZD7442, a monoclonal antibody cocktail, in a new arm of the phase III trial.

## 4. Discussion

Belgian hospitals, particularly those more usually involved in clinical research, took part in clinical studies from the very early days of the pandemic to try to identify effective therapeutic options against COVID-19 in hospitalized patients. Nevertheless, less than 3% of patients hospitalized with COVID-19 in Belgium participated in clinical research during this same period, when the whole world was scrambling to identify effective therapeutics. This number contrasts with the one in six patients hospitalized for COVID-19 included in the UK in the RECOVERY trial. Although hospitals were overwhelmed, and a certain amount of expertise is needed to carry out clinical research of quality, centers could have participated in a more pragmatic trial such as EU-SolidAct (https://eu-response.eu/eu-solidact/) (Accessed on 24 June 2022), which allows centers to participate with different levels of involvement (e.g., participation without taking samples for biobanking). Patients in Belgium are often hospitalized in an institution close to their home. This means that if a patient did not live near a university hospital or one with a university link, their chances of participating in a clinical trial on COVID-19 therapeutics to help find a cure for the disease, but also to possibly benefit at an individual level from a therapy being evaluated, were almost null.

Among the 28 trials carried out, only four had an impact on recommendations for therapeutic guidance: the REMAP-CAP, the DisCoVeRy, the DAWN-plasma, and the COV-AID trials. All four of these trials had more than 15 clinical trial sites and/or were adaptive platform trials. Adaptive platform trials enable simultaneous assessment of multiple interventions, while allowing interventional arms to be dropped as evidence becomes available. REMAP-CAP helped to provide guidance on the use of corticosteroids [13], anticoagulation treatment [14], interleukin (IL)-6 [15] and IL-1 blockers [16], and convalescent plasma [17]; the DisCoVeRy trial gave guidance on treatment with hydroxychloroquine, lopinavir–ritonavir, lopinavir–ritonavir plus interferon ß-1a [7,18], and remdesivir [7,19]; the DAWN trial provided guidance on the use of convalescent plasma [20]; and the COV-AID trial gave guidance on the administration of IL-1 and IL-6 blockers [21]. Nevertheless, despite the numerous Belgian investigation sites (>15) for the COV-AID and DAWN-plasma trials, it took several waves of the pandemic to complete recruitment for both trials, with a total recruitment of 342 patients and 483 patients, respectively. Results for these trials were therefore obtained after other larger, international trials obtained results concerning the same investigational products [15,17,22,23] (even when evaluation of these investigational products was initiated after evaluation had started in Belgium). Multicenter international trials had the advantage of being able to recruit patients in a continuous, steady fashion because when the pandemic was waning in one country, it was increasing in another. 

Approximately one fifth of the trials in Belgium were small, mono- or bicenter studies. Most of these smaller trials initiated at the beginning of the pandemic were terminated early because conclusive results showing the futility of the drugs being evaluated were obtained once again much faster by larger, international platform trials. These smaller trials consumed considerable time and energy without providing additional useful scientific knowledge to help provide guidance on therapeutics for COVID-19, during a period when time, energy, and money were precious commodities. 

Of course, general trial recruitment could have been more rapid if patient participation in Belgium had been greater. Indeed, less than 3% of all patients hospitalized with COVID-19 participated in a clinical trial on therapeutics. This can unfortunately be contrasted with the over 4500 patients who received off-label hydroxychloroquine during the first wave of the pandemic [24], and the over 2500 treatments of remdesivir administered to patients until the 31 January 2021. During this same period, the WHO [25] and Belgian guidelines for COVID-19 in hospitalized patients recommended against the administration of remdesivir in hospitalized patients, except in the context of a clinical trial. Drug consumption data concerning IL-6 blockers and tofacitinib via compassionate use programs are not available. However, the story is similar to those of remdesivir and hydroxychloroquine. While recruitment was ongoing for the COV-AID trial and the EU-SolidAct trial, patients received compassionate use of IL-6 blockers and of tofacitinib, respectively, despite a lack of evidence showing the clear efficacy of either drug in hospitalized patients with COVID-19. This illustrates that there is a lack of “clinical trial culture” in the general Belgian population, but also among healthcare workers, with people more open to receiving or prescribing (in the case of a doctor) off-label drugs than participating in clinical trials, even when there is a lack of clear evidence supporting treatment efficacy. Further education of healthcare workers and the general public on the importance of clinical trials, particularly during a pandemic period, when effective therapies for the disease are unknown, is essential.

Other countries have faced similar challenges. Evaluation of Canada’s research response to COVID-19 showed that the research structures were inefficient, that efforts were fragmented, and that clinical research and clinical care were too separate [26]. Assessment of the clinical research response to COVID-19 in the USA also identified inefficient clinical research structures, limited incentives for collaboration, lack of prioritization from a minimally regulated market-based system, and lack of integrated coordination between research and clinical care [27]. On the other hand, the UK was able to adopt streamlined and pragmatic procedures, accompanied by widespread collaboration focused on the single goal of identifying a safe and effective therapy for COVID-19 via the RECOVERY trial. This trial, with the impressive number of patients recruited and scientific results obtained, based on the collection of routine healthcare data, illustrates how clinical trials and healthcare can be integrated during a pandemic, despite an overstretched healthcare system [28].

On a positive note, ethical and regulatory approvals for the different COVID-19 trials were obtained quite rapidly in Belgium. A comparison with other countries in Europe concerning the time needed to obtain the different regulatory approvals had already shown that Belgium was faster than many other European countries to provide approvals for the DisCoVeRy trial [29]. 

Concerning the financing of the COVID-19 clinical trials, federal money was made available very rapidly at the beginning of the pandemic to finance trials on COVID-19 therapeutics. However, the KCE trial rules do not permit financing of trials that evaluate drugs which have not yet received marketing authorization. Although this rule is defendable when not in a pandemic situation, it is problematic during a pandemic, when repurposed drugs were evaluated, and no effective antivirals had been identified. Indeed, after the repurposed drugs were shown not to be effective, academic trials wanted to evaluate promising drugs in the pipeline for COVID-19. Because of this rule, the DisCoVeRy trial lost KCE funding when it began to evaluate the AZD7442 arm for efficacy and safety. The Belgium DisCoVeRy team then had to spend time and energy looking for another source of financing. It is true that the DisCoVeRy trial is now partially financed by EU Horizon 2020, which has allocated EUR 15.7 million to the EU-Response project for this purpose. Although this is significant funding, the EU-Response project is made up of four work-packages, two of which are clinical trials. It is planned that these clinical trials will both open in over 14 different European countries, at over 90 different sites (https://eu-response.eu) (Accessed 24 June 2020)—EUR 15.7 million will not cover the costs of the entire project, and financial support to develop a sustainable European adaptive clinical trial platform for emerging infectious diseases from member European states is still necessary. 

## 5. Conclusions 

Belgium has actively participated in clinical research efforts to identify safe and effective therapeutics for hospitalized patients with COVID-19 since the beginning of the pandemic. Nevertheless, as with many other countries, more effort could have been made to avoid running small, under-powered, mono- or bicenter trials, to create better collaboration between the different Belgian hospitals, and to participate in more international clinical trials, and more specifically in adaptive platform trials. The international collaboration is particularly relevant because Belgium is a small country. When considering platform trials, Belgium could take the lead for one of the trial arms, meaning that a Belgian center could participate actively in the trial’s steering committee. Another option would be to invest in developing nationwide electronic health record data for trial outcome assessments; this would allow for easy implementation of pragmatic clinical trials, as was done in the RECOVERY trial. This could facilitate greater participation of general hospitals in clinical research, even after the COVID-19 pandemic is over. 

Energy should also be invested in educating caregivers and the general population on the importance of clinical trials. Significantly greater participation from all players (patients and caregivers) is still needed. Improving participation in clinical trials could indeed be an asset during non-pandemic times. Finally, rules concerning the financing of trials should not be followed so stringently during a pandemic. Phase III trials of drugs that do not have marketing authorization are scientifically important when a pandemic has evolved and repurposing of drugs is no longer attractive as a therapeutic solution. However, large academic trials need adequate funding.

## Figures and Tables

**Table 1:** Registered industry-sponsored clinical trials on COVID-19 therapeutics conducted in hospitalized patients in Belgium between 1 March 2020 and 31 December 2021 (with at least one patient included) *.

Trial	Trial Registration Number	Study Design	Number of Patients Included	Placebo Controlled	Duration for Ethics and Regulatory Approvals	Patient Population	Date of Study Initiation to Date of Termination/Completion	If Terminated or Completed, Publication or Communication of Results
Clinical trial to assess the efficacy and safety of inhaled AQ001S in the management of acute COVID-19 symptoms (SIROCCO-1)	NCT05000346	Phase II, RCT	99	Yes	Not available	Non-ICU	4/11/2021–ongoing	Not applicable
A randomized, double-blind, placebo-controlled trial to determine the safety and efficacy of estetrol (E4) for the treatment of patients with confirmed SARS-CoV-2 infection	2020-003403-33	Phase II, RCT	50 in Belgium, 300 in total	Yes	36 days	Non-ICU	20/01/2021– 24/9/2021 Ended for futility	No
Exploratory study of the safety, tolerability and efficacy of nangibotide in patients with COVID-19 receiving ventilator support and features of systemic inflammation—A randomized, double-blind, placebo-controlled study with adaptive features	2020-001504-42	Phase II, RCT	20 in Belgium, 60 in total	Yes	37 days	ICU	15/7/2020–ongoing	Not applicable
A randomized, open-label, multicenter, phase 2a study to evaluate the safety and effect of STC3141 continuous infusion in subjects with severe COVID-19 pneumonia	2021-000399-12	Phase II, open label	25	No	25 days	Non-ICU + ICU	16/4/2021–07/01/22	No
A randomized, double-blind, placebo-controlled, phase III trial to determine the efficacy and safety of inhaled SNG001 for the treatment of patients hospitalized due to moderate COVID-19	2020-004743-83	Phase III, RCT	35 in Belgium, 610 in total	Yes	60 days	Non-ICU	9/11/2020– 10/2/2022	No
A multicenter, open-label, pharmacokinetic and safety study of baricitinib in pediatric patients from 1 year to less than 18 years old hospitalized with COVID-19	2021-001338-21	Phase III, open label	8 in Belgium, 24 in total	No	28 days	Non-ICU + ICU	4/11/2021–ongoing	Not applicable
Evaluation of the efficacy and safety of PTC299 in hospitalized subjects with COVID-19 (FITE19)	2020-001872-13	Phase II/III RCT	20 in Belgium, 380 in total	Yes	1 day	Non-ICU	09/07/20–30/6/22	No
Adaptive design phase 2 to 3, randomized, double-blind, multicenter, to evaluate the safety, efficacy, pharmacokinetics and pharmacodynamics of BI0101 in the prevention of the respiratory deterioration in hospitalized patients with COVID-19 pneumonia (severe stage)	2020-001498-63	Phase II, RCT	65 in Belgium 465 in total	Yes	15 days	Non-ICU	22/7/2020–ongoing	Not applicable
A randomized, double-blind, placebo-controlled study evaluating the efficacy and safety of otilimab IV in patients with severe pulmonary COVID-19-related disease	2020-001759-42	Phase II/III	4 in Belgium, 1156 in total	Yes	16 days	ICU	19/6/2020–16/8/2021	No
The TRISTARDS trial—ThRombolysiS Therapy for ARDS A Phase II/III operationally seamless, open-label, randomized, sequential, parallel-group adaptive study to evaluate the efficacy and safety of daily intravenous alteplase treatment given up to 5 days on top of SOC compared with SOC alone, in patients with ARDS triggered by COVID-19	2020-002913-16	Phase II	6 in Belgium, 270 in total	No	21 days	ICU	26/11/2021–ongoing	Not applicable
First-in-human study to evaluate safety, tolerability, and pharmacokinetics following single ascending and multiple ascending doses of PF-07304814 in hospitalized participants with COVID-19	NCT04535167	Phase 1b, two-part, double-blind, placebo-controlled	26 in total	Yes	Unknown	Non-ICU	09/09/2020–07/06/2021	No
A pragmatic adaptive randomized, controlled phase II/III multicenter study of IFX-1 in patients with severe COVID-19 (PANAMO)	2020-001335-28	Phase II/III	19 Belgium, 390 in total	Yes	15 days	Non-ICU + ICU	31/03/2020–1/12/2021	No
A 2-part clinical study including a first-in-human, open-label, single ascending dose part (phase I) followed by a randomized, double-blind, placebo-controlled part (phase II) to evaluate the efficacy and safety of XVR011 in patients hospitalized for mild to moderate COVID-19	2020-005299-36	Phase I/II: randomized, double-blind, placebo-controlled trial	50 in Belgium, 279 in total	Yes	Unknown days	Non-ICU	26/08/2021–18/3/2022 (terminated early)	No

* Table completed using data available on 11 May, 2022; RCT: randomized controlled trial.

**Table 2:** Registered academic clinical trials on COVID-19 therapeutics conducted in hospitalized patients in Belgium between 1 March 2020 and 31 December 2021 (with at least one patient included) *.

Trial	Trial Registration Number	Study Design	Number of Patients Included	Placebo Controlled	Duration for Ethics and Regulatory Approvals	Patient Population	Date of Study Initiation to Date of Termination/Completion	If Terminated or Completed, Publication or Communication of Results
A prospective, randomized, open-label, interventional study to investigate the efficacy of sargramostim (Leukine^®^) in improving oxygenation and short- and long-term outcome of COVID-19 patients with acute hypoxic respiratory failure	2020-001254-22	Phase IV, RCT open-label	80	No	1 day	Non-ICU + ICU (but patients needing mechanical ventilation were excluded)	24/3/2020–26/2/2021 Completed	No
COVID-19: A randomized, open-label, adaptive, proof-of-concept clinical trial of new antiviral drug candidates against SARS-CoV-2	2020-001243-15	Phase II, RCT open-label	68 enrolled, 200 planned	No	2 days	Non-ICU	26/3/2020–10/6/2020 Prematurely ended	Yes
Multicenter, adaptive, randomized trial of the safety and efficacy of treatments of COVID-19 in hospitalized adults (DisCoVeRy)	2020-00936-23	Phase III, RCT, adaptive platform trial	51 in Disco1 ^a^ 10 for Disco2	No in Disco1 Yes in Disco2	54 days	Disco1: Non-ICU + ICU Disco2: Non-ICU	20/5/2020–ongoing	DisCoVeRy1: Yes DisCoVeRy2: Not applicable
A randomized, open-label, adaptive, proof-of-concept clinical trial of modulation of host thromboinflammatory response in patients with COVID-19	2020-001739-28	Phase II, RCT	210	No	40 days	Non-ICU + ICU	20/5/2020–5/4/2021	No
A prospective, randomized, factorial design, interventional study to compare the safety and efficacy of combinations of blockade of interleukin-6 pathway and interleukin-1 pathway to best standard of care in improving oxygenation and short- and long-term outcome of COVID-19 patients with acute hypoxic respiratory failure and systemic cytokine release syndrome (COV-AID) ^a^	2020-001500-41	Phase III, RCT open-label	342	No	1 day	Non-ICU and ICU	3/4/2020–21/5/2021	Yes
COVID-19: A randomized, open-label, adaptive, proof-of-concept clinical trial of new antiviral drug candidates against SARS-CoV-2	2020-001614-38	Phase II, open-label	185 out of 282 planned patients	No	16 days	Non-ICU	22/4/2020–17/12/2020, prematurely ended	Yes
COVID-19: Experimental use of tociluzimab (Roactemra^®^) in severe SARS-CoV-2-related pneumonia	2020-001770-30	Phase II, RCT, open-label	60	No	6 days	Non-ICU + ICU	21/4/2020–unknown, but prematurely ended	No
DAWN-plasma ^a^	NCT04429854	Phase II, RCT, open-label	483	No	Unknown	Non-ICU + ICU	2/5/2020	Yes
Randomized, embedded, multifactorial, adaptive platform trial for community-acquired pneumonia (REMAP-CAP)—COVID-19 patients included	2015-002340-14	Phase III, RCT, adaptive, platform trial	250 planned in Belgium, 17,802 patients included globally until 11/5/22	No	Unknown	ICU	9/3/2020–ongoing	Yes, of completed arms
A prospective, randomized, open-label, interventional study to investigate the efficacy of complement C5 inhibition with Zilcoplan^®^ in improving oxygenation and short- and long-term outcome of COVID-19 patients with acute hypoxic respiratory failure (ZILU-COV)	2020-002130-33	Phase II, RCT	81	No	20 days	Non-ICU + ICU	26/5/2021–completed	No
A multicenter, randomized trial to assess the efficacy of CONvalescent Plasma Therapy in patients with invasive COVID-19 and acute respiratory failure treated with mechanical ventilation: the CONFIDENT Trial ^a^	NCT04558476	Phase II, RCT	500 (475 currently included)	No	Unknown	ICU	1/9/2020–ongoing	No
Mesenchymal stromal cell therapy for severe COVID-19 infection	2020-002102-58	Phase II, RCT, Open-label	20	No	6 days	Non-ICU + ICU	12/6/2020–ongoing	Not applicable
Pulsed, inhaled nitric oxide (iNO) for the treatment of patients with mild or moderate COVID-19	2020-002394-94	Phase II, RCT, open-label	6	No	18 days	Non-ICU	22/4/2020–ongoing	Not applicable
Alkaline phosphate for reducing inflammatory syndrome (SIRS) in patients with SARS-CoV-2 infection and acute respiratory insufficiency (COVID-19)	2020-001714-38	Phase II, RCT	44 in Belgium, 132 in trial	Yes	64 days	Non-ICU + ICU	7/6/2021–ongoing	Not applicable
European DisCoVeRy for Solidarity: An adaptive pandemic and emerging infection platform trial	2021-000541-41	Phase III, RCT, adaptive platform trial	6 in Belgium	Yes	2 days for regulatory approval	Non-ICU + ICU	8/10/2021–ongoing	Not applicable

* Table completed using data available on 11 May 2022; ^a^ KCE-funded trial; RCT: randomized, controlled trial; ICU: intensive care unit.

## Data Availability

Not applicable.

## References

[1] World Health Organization WHO Coronavirus (COVID-19) Dashboard. https://covid19.who.int/?mapFilter=deaths.

[2] Thielman N.M., Cunningham C.K., Woods C., Petzold E., Sprenz M., Russell J. (2016). Ebola clinical trials: Five lessons learned and a way forward. Clin. Trials.

[3] WHO Ethics Working Group Meeting. Ethical Issues Related to Study Design for Trials on Therapeutics for Ebola Virus Disease. https://apps.who.int/iris/handle/10665/137509.

[4] Saville B.R., Berry S.M. (2016). Efficiencies of platform clinical trials: A vision of the future. Clin. Trials.

[5] Sacks C.A., North C.M., Wolf M., Dougan M., Campbell K.R., Moggridge J., Fralick M. (2022). The landscape of COVID-19 research in the United States: A cross-sectional study of randomized trials registered on ClinicalTrials.Gov. J. Gen. Intern. Med..

[6] Gerkens S., Merkur S. (2020). Belgium: Health System Review. Health Syst. Transit..

[7] Pan H., Peto R., Henao-Restrepo A.M., Preziosi M.P., Sathiyamoorthy V., Abdool K.Q., Alejandria M.M., Hernández G., Kieny M.P., Malekzadeh R. (2021). Repurposed antiviral drugs for COVID-19—Interim WHO Solidarity Trial Results. N. Engl. J. Med..

[8] Shankar-Hari M., Vale C.L., Godolphin P.J., Fisher D., Higgins J.P.T., Spiga F., Savovic J., Tierney J., Baron G., Benbenishta J.S. (2021). Association between administration of IL-6 antagonists and mortality among patients hospitalized for COVID-19: A meta-analysis. JAMA.

[9] Sterne J.A.C., Murthy S., Diaz J.V., Slutsky A.S., Villar J., Angus D.C., Annane D., Azevedo L.C.P., Berwanger O., Cavalcanti A.B. (2020). Association between administration of systemic corticosteroids and mortality among critically ill patients with COVID-19: A meta-analysis. JAMA.

[10] Axfors C., Janiaud P., Schmitt A.M., Hooft J.V., Smith E.R., Haber N.A., Abayomi A., Abduljalil M., Abdulrahman A., Acosta-Ampudia Y. (2021). Association between convalescent plasma treatment and mortality in COVID-19: A collaborative systematic review and meta-analysis of randomized clinical trials. BMC Infect. Dis..

[11] Sciensano Belgium COVID-19 Epidemiological Situation. https://datastudio.google.com/embed/u/0/reporting/c14a5cfc-cab7-4812-848c-0369173148ab/page/uTSKB.

[12] Belgian Health Care Knowledge Centre Annual Report 2020. https://annualreport.kce.be/2020/PDF/EN/annual_report_2020.pdf.

[13] Angus D.C., Derde L., Al-Beidh F., Annane D., Arabi Y., Beane A., van Bentum-Puijk W., Berry L., Bhimani Z., Bonten M. (2020). Effect of hydrocortisone on mortality and organ support in patients with severe COVID-19: The REMAP-CAP COVID-19 Corticosteroid Domain Randomized Clinical Trial. JAMA.

[14] Goligher E.C., Bradbury C.A., McVerry B.J., Lawler P.R., Berger J.S., Gong M.N., Carrier M., Reynolds H.R., Kumar A., Turgeon A.F. (2021). Therapeutic anticoagulation with heparin in critically ill patients with COVID-19. N. Engl. J. Med..

[15] Gordon A.C., Mouncey P.R., Al-Beidh F., Rowan K.M., Nichol A.D., Arabi Y.M., Annane D., Beane A., van Bentum-Puijk W., Berry L.R. (2021). Interleukin-6 receptor antagonists in critically ill patients with COVID-19. N. Engl. J. Med..

[16] REMAP-CAP Investigators. Derde LPG (2021). Effectiveness of tocilizumab, sarilumab, and anakinra for critically ill patients with COVID-19 The REMAP-CAP COVID-19 Immune Modulation Therapy Domain Randomized Clinical Trial. medRxiv.

[17] Estcourt L.J., Turgeon A.F., McQuilten Z.K., McVerry B.J., Al-Beidh F., Annane D., Arabi Y.M., Arnold D.M., Beane A., Bégin P. (2021). Effect of convalescent plasma on organ support-free days in critically Ill patients with COVID-19: A randomized clinical trial. JAMA.

[18] Ader F., Peiffer-Smadja N., Poissy J., Bouscambert-Duchamp M., Belhadi D., Diallo A., Delmas C., Saillard J., Dechanet A., Mercier N. (2021). An open-label randomized controlled trial of the effect of lopinavir/ritonavir, lopinavir/ritonavir plus IFN-beta-1a and hydroxychloroquine in hospitalized patients with COVID-19. Clin. Microbiol. Infect..

[19] Ader F., Bouscambert-Duchamp M., Hites M., Peiffer-Smadja N., Poissy J., Belhadi D., Diallo A., Lê M.-P., Peytavin G., Staub T. (2021). Remdesivir plus standard of care versus standard of care alone for the treatment of patients admitted to hospital with COVID-19 (DisCoVeRy): A phase 3, randomised, controlled, open-label trial. Lancet Infect. Dis..

[20] Devos T., Van Thillo Q., Compernolle V., Najdovski T., Romano M., Dauby N., Jadot L., Leys M., Maillart E., Loof S. (2021). Early high antibody titre convalescent plasma for hospitalised COVID-19 patients: DAWn-plasma. Eur. Respir. J..

[21] Declercq J.A., Van Damme K.F., De Leeuw E., Maes B., Bosteels C., Tavernier S.J., De Buyser S., Colman R., Hites M., Verschelden G. (2021). Effect of anti-interleukin drugs in patients with COVID-19 and signs of cytokine release syndrome (COV-AID): A factorial, randomised, controlled trial. Lancet Respir. Med..

[22] RECOVERY Collaborative Group (2021). Convalescent plasma in patients admitted to hospital with COVID-19 (RECOVERY): A randomised controlled, open-label, platform trial. Lancet.

[23] RECOVERY Collaborative Group (2021). Tocilizumab in patients admitted to hospital with COVID-19 (RECOVERY): A randomised, controlled, open-label, platform trial. Lancet.

[24] Catteau L., Dauby N., Montourcy M., Bottieau E., Hautekiet J., Goetghebeur E., van Ierssel S., Duysburgh E., Van Oyen H., Wyndham-Thomas C. (2020). Low-dose hydroxychloroquine therapy and mortality in hospitalised patients with COVID-19: A nationwide observational study of 8075 participants. Int. J. Antimicrob. Agents.

[25] World Health Organization Therapeutics and COVID-19. https://apps.who.int/iris/bitstream/handle/10665/340374/WHO-2019-nCoV-therapeutics-2021.1-eng.pdf?sequence=1&isAllowed=y.

[26] Lamontagne F., Rowan K.M., Guyatt G. (2020). Integrating research into clinical practice: Challenges and solutions for Canada. Can. Med. Assoc. J..

[27] Angus D.C., Gordon A.C., Bauchner H. (2021). Emerging Lessons From COVID-19 for the US Clinical Research Enterprise. JAMA J. Am. Med. Assoc..

[28] Pessoa-Amorim G., Campbell M., Fletcher L., Horby P., Landray M., Mafham M., Haynes R. (2021). Making trials part of good clinical care: Lessons from the RECOVERY trial. Future Health J..

[29] Diallo A., Trøseid M., Simensen V.C., Boston A., Demotes J., Olsen I.C., Chung F., Paiva J.A., Hites M., Ader F. (2021). Accelerating clinical trial implementation in the context of the COVID-19 pandemic: Challenges, lessons learned and recommendations from DisCoVeRy and the EU-SolidAct EU response group. Clin. Microbiol. Infect..

